# Supplementary material for: COVID-19 convalescent plasma for B-cell depleted patients: a systematic review and individual patient data meta-analysis
Source: Front Med (Lausanne). 2025 Oct 17;12:1658593. doi: 10.3389/fmed.2025.1658593 (PMC12575326; doi:10.3389/fmed.2025.1658593)
Supplement: Supplementary file 1 [file Data_Sheet_1.pdf]

Supplementary Appendix

This appendix has been provided by the authors to give readers additional information about their work.

Supplement to: Convalescent plasma treatment of B-cell depleted patients with COVID-19: systematic review and individual participant data meta-analysis.

CONTENTS

Table S1. Patient characteristics according to year of infection. 3

Table S2. Treatment characteristics according to year of infection. 4

Table S3. Outcomes according to year of infection. 5

Table S4. Risk of bias assessment of randomized controlled trial. 6

Table S5. Risk of bias assessment of non-randomized controlled trial. 9

Table S6. Risk of bias assessment of cohort studies. 12

Table S7. Risk of bias assessment of case series. 13

Table S8. Risk of bias assessment of case reports. 15

Table S9. World Health Organization (WHO) disease severity scale for COVID-19. 17

Table S10. PRISMA Individual Participant Data meta-analysis checklist 2020. 18

Figure S1. Receiver operating characteristic curve from final XGBoost model for predicting 60-day survival. 23

Table S1. Patient characteristics according to year of infection.

Characteristic	2020 (N=246)		2021 (N=115)		2022/2023 (N=209)	
	N	Summary	N	Summary	N	Summary
Patient type	245		113		209	
Autoimmune disorder		30 (12.2%)		18 (15.9%)		66 (31.6%)
Hematologic malignancy/disorder		214 (87.3%)		95 (84.1%)		141 (67.5%)
Solid organ transplantation		1 (0.4%)		0 (0.0%)		2 (1.0%)
Age, years	242	62 (52, 70)	114	62 (53, 72)	209	66 (56, 74)
Sex	245		115		209	
Female		98 (40.0%)		42 (36.5%)		92 (44.0%)
Male		147 (60.0%)		73 (63.5%)		117 (56.0%)
Days since the last anti-CD20 dose	187	53 (23, 120)	75	46 (13, 106)	131	67 (25, 152)
Mechanical ventilation	239	40 (16.7%)	113	16 (14.2%)	208	11 (5.3%)
WHO Disease Severity ≥ 5	241	93 (38.6%)	113	39 (34.5%)	208	37 (17.8%)
Number of COVID-19 vaccine doses	197		97		199	
0		155 (78.7%)		55 (56.7%)		12 (6.0%)
1		1 (0.5%)		9 (9.3%)		3 (1.5%)
2		26 (13.2%)		22 (22.7%)		49 (24.6%)
3		15 (7.6%)		9 (9.3%)		68 (34.2%)
4 or more		0 (0.0%)		2 (2.1%)		67 (33.7%)
Anti-spike Ab status	161		77		103	
Negative		151 (93.8%)		67 (87.0%)		88 (85.4%)
Positive		10 (6.2%)		10 (13.0%)		15 (14.6%)

Data are summarized using median (25th, 75th) for continuous variables and n (%) for categorical variables.
WHO, World Health Organization. Ab, antibody.

Table S2. Treatment characteristics according to year of infection.

Characteristic	2020 (N=246)		2021 (N=115)		2022/2023 (N=209)	
	N	Summary	N	Summary	N	Summary
Treatment of underlying condition						
BTK inhibitors	239	5 (2.1%)	103	4 (3.9%)	198	17 (8.6%)
Anti-CD19 CAR T-cell therapy	221	4 (1.8%)	103	3 (2.9%)	202	5 (2.5%)
Bendamustine	220	37 (16.8%)	100	14 (14.0%)	201	31 (15.4%)
Venetoclax	220	9 (4.1%)	101	5 (5.0%)	200	11 (5.5%)
CCP treatment						
Type of plasma transfused	246		115		209	
CCP or Vaccine-only plasma		237 (96.3%)		105 (91.3%)		99 (47.4%)
Vaccine-boosted CCP		9 (3.7%)		10 (8.7%)		110 (52.6%)
Total units transfused, units	234	3 (2, 4)	105	3 (2, 4)	206	3 (2, 4)
Total volume transfused, mL	234	713 (400, 800)	105	800 (450, 840)	206	720 (360, 800)
Days from first symptoms*	190	31 (17, 56)	90	13 (7, 24)	192	9 (2, 23)
Days from hospital admission*	147	16 (6, 38)	59	5 (2, 12)	51	6 (4, 18)
Concomitant COVID-19 therapy						
Non-specific IVIG	182	25 (13.7%)	90	9 (10.0%)	199	13 (6.5%)
Steroids	195	138 (70.8%)	90	68 (75.6%)	197	96 (48.7%)
Remdesivir	201	81 (40.3%)	104	51 (49.0%)	201	86 (42.8%)
Nirmatrelvir/ritonavir	199	0 (0.0%)	97	0 (0.0%)	198	43 (21.7%)
Hydroxychloroquine	203	22 (10.8%)	0	0 (0.0%)	0	0 (0.0%)
Anti-spike mAb	202	6 (3.0%)	99	6 (6.1%)	201	70 (34.8%)

Data are summarized using median (25th, 75th) for continuous variables and n (%) for categorical variables.
*Days to first plasma transfusion.
BTK, Bruton tyrosine kinase. CAR, chimeric antigen receptor. CCP, COVID-19 convalescent plasma. IVIG, intravenous immunoglobulin. mAb, monoclonal antibodies.

Table S3. Outcomes according to year of infection.

Characteristic	2020 (N=246)		2021 (N=115)		2022/2023 (N=209)	
	N	Summary	N	Summary	N	Summary
60-day survival	246	209 (85.0%)	115	96 (83.5%)	209	188 (90.0%)
Rapid improvement within 5 days	177	107 (60.5%)	79	47 (59.5%)	189	132 (69.8%)
SARS-CoV-2 clearance	194	132 (68.0%)	100	75 (75.0%)	117	81 (69.2%)

Data are summarized using n (%).

Table S4. Risk of bias assessment of randomized controlled trial.

Risk of bias for Denkinger et al. trial	Yes	No	Unclear	N/A
Bias related to selection and allocation				
1. Was true randomization used for assignment of participants to treatment groups?	■	□	□	□
2. Was allocation to treatment groups concealed?	■	□	□	□
3. Were treatment groups similar at the baseline?	□	□	■	□
Bias related to administration of intervention/exposure				
4. Were participants blind to treatment assignment?	□	■	□	□
5. Were those delivering the treatment blind to treatment assignment?	□	■	□	□
6. Were treatment groups treated identically other than the intervention of interest?	■	□	□	□
Bias related to assessment, detection and measurement of the outcome				
7. Were outcome assessors blind to treatment assignment?				
Outcome 1	□	■	□	□
Outcome 2	□	■	□	□
Outcome 3	□	■	□	□
Outcome 4	□	■	□	□
Outcome 5	□	■	□	□
8. Were outcomes measured in the same way for treatment groups?				
Outcome 1	■	□	□	□
Outcome 2	■	□	□	□

Outcome 3	■	□	□	□
Outcome 4	■	□	□	□
Outcome 5	■	□	□	□
9. Were outcomes measured in a reliable way				
Outcome 1	■	□	□	□
Outcome 2	■	□	□	□
Outcome 3	■	□	□	□
Outcome 4	■	□	□	□
Outcome 5	■	□	□	□
Bias related to participant retention				
10. Was follow up complete and if not, were differences between groups in terms of their follow up adequately described and analyzed?				
Outcome 1	■	□	□	□
Outcome 2	■	□	□	□
Outcome 3	■	□	□	□
Outcome 4	■	□	□	□
Outcome 5	■	□	□	□
Statistical Conclusion Validity				
11. Were participants analyzed in the groups to which they were randomized?				
Outcome 1	■	□	□	□
Outcome 2	■	□	□	□

Outcome 3	■	□	□	□
Outcome 4	■	□	□	□
Outcome 5	■	□	□	□
12. Was appropriate statistical analysis used?				
Outcome 1	■	□	□	□
Outcome 2	■	□	□	□
Outcome 3	■	□	□	□
Outcome 4	■	□	□	□
Outcome 5	■	□	□	□
13. Was the trial design appropriate and any deviations from the standard RCT design (individual randomization, parallel groups) accounted for in the conduct and analysis of the trial?	■	□	□	□

Overall risk of bias: Low risk

Table S5. Risk of bias assessment of non-randomized controlled trial.

Risk of bias for Marconato et al. trial	Yes	No	Unclear	N/A
Bias related to temporal precedence				
1. Is it clear in the study what is the “cause” and what is the “effect” (i.e. there is no confusion about which variable comes first)?	■	☐	☐	☐
Bias related to selection and allocation				
2. Was there a control group?	☐	■	☐	☐
Bias related to confounding factors				
3. Were participants included in any comparisons similar?	☐	☐	☐	■
Bias related to administration of intervention/exposure				
4. Were the participants included in any comparisons receiving similar treatment/care, other than the exposure or intervention of interest?	☐	☐	☐	■
5. Were there multiple measurements of the outcome, both pre and post the intervention/exposure?				
Outcome 1	■	☐	☐	☐
Outcome 2	■	☐	☐	☐
Outcome 3	■	☐	☐	☐
Outcome 4	■	☐	☐	☐
Outcome 5	■	☐	☐	☐

6. Were the outcomes of participants included in any comparisons measured in the same way?				
Outcome 1	■	□	□	□
Outcome 2	■	□	□	□
Outcome 3	■	□	□	□
Outcome 4	■	□	□	□
Outcome 5	■	□	□	□
7. Were outcomes measured in a reliable way?				
Outcome 1	■	□	□	□
Outcome 2	■	□	□	□
Outcome 3	■	□	□	□
Outcome 4	■	□	□	□
Outcome 5	■	□	□	□
Bias related to participant retention				
8. Was follow-up complete and if not, were differences between groups in terms of their follow-up adequately described and analyzed?				
Outcome 1	■	□	□	□
Outcome 2	■	□	□	□
Outcome 3	■	□	□	□

Outcome 4	■	□	□	□
Outcome 5	■	□	□	□
Statistical Conclusion Validity				
9. Was appropriate statistical analysis used?				
Outcome 1	□	□	□	■
Outcome 2	■	□	□	□
Outcome 3	■	□	□	□
Outcome 4	■	□	□	□
Outcome 5	■	□	□	□
**Overall risk of bias: Low risk**				

Table S6. Risk of bias assessment of cohort studies.

Study	Risk of bias assessment tool questions											Overall risk
	Q1	Q2	Q3	Q4	Q5	Q6	Q7	Q8	Q9	Q10	Q11	
Aiello et al, 2024	N/A	N/A	Yes	Yes	No	Yes	Yes	Yes	No	Unclear	Yes	Moderate
Brown et al, 2022	N/A	N/A	Yes	Yes	No	Yes	Unclear	Unclear	Unclear	N/A	Unclear	High
Destremau et al, 2024	N/A	N/A	Yes	Yes	Yes	Yes	Yes	Yes	Yes	N/A	Yes	Low
Hueso et al, 2022	N/A	N/A	Yes	Yes	Yes	Yes	Yes	Yes	Yes	N/A	Yes	Low
Ioannou et al, 2023	N/A	N/A	Yes	Yes	Yes	Yes	Yes	Yes	Unclear	Unclear	Yes	Low
Lanza et al, 2022	Yes	Yes	Yes	Yes	Yes	Yes	Yes	Yes	Yes	N/A	Yes	Low
Magyari et al, 2022	N/A	N/A	Yes	Yes	No	Yes	Yes	Yes	Yes	N/A	Yes	Low
Richier et al, 2024	N/A	N/A	Yes	Unclear	Unclear	Yes	Yes	Yes	Yes	N/A	Yes	Low
Thümmler et al, 2022	No	Yes	Yes	Yes	Yes	Yes	Yes	Yes	Yes	N/A	Yes	Low
Weinbergerová et al, 2022	Unclear	Yes	Yes	Yes	No	Yes	Yes	Unclear	Unclear	Unclear	Yes	Moderate
Weisser et al, 2022	Yes	Yes	Yes	Unclear	Yes	Yes	Yes	Yes	Yes	Yes	Yes	Low

Q1: Were the two groups similar and recruited from the same population? Q2: Were the exposures measured similarly to assign people to both exposed and unexposed groups? Q3: Was the exposure measured in a valid and reliable way? Q4: Were confounding factors identified? Q5: Were strategies to deal with confounding factors stated? Q6: Were the groups/participants free of the outcome at the start of the study (or at the moment of exposure)? Q7: Were the outcomes measured in a valid and reliable way? Q8: Was the follow up time reported and sufficient to be long enough for outcomes to occur? Q9: Was follow up complete, and if not, were the reasons to loss to follow up described and explored? Q10: Were strategies to address incomplete follow up utilized? Q11: Was appropriate statistical analysis used?
Overall risk of bias was scored in the following way: ≤49% yes = high risk of bias, 50-69% yes = moderate risk of bias, ≥70% yes = low risk of bias.

Table S7. Risk of bias assessment of case series.

Study	Risk of bias assessment tool questions										Overall risk
	Q1	Q2	Q3	Q4	Q5	Q6	Q7	Q8	Q9	Q10	
Bronstein et al, 2021	Yes	Yes	Yes	Unclear	Unclear	Yes	Yes	Yes	Yes	N/A	Low
Cusi et al, 2021	Yes	Yes	Yes	Unclear	Unclear	Yes	Yes	Yes	Yes	N/A	Low
D'Abramo et al, 2022	Yes	Yes	Yes	Yes	Unclear	Yes	Yes	Yes	Yes	N/A	Low
Da Silva et al, 2023	Yes	Unclear	Unclear	Unclear	Unclear	Yes	Yes	Yes	Unclear	N/A	High
Delgado-Fernández et al, 2022	Yes	Yes	Yes	Yes	Yes	Yes	Yes	Yes	Yes	N/A	Low
Deveci et al, 2021	Yes	Yes	Yes	Unclear	Unclear	Yes	Yes	Yes	No	N/A	Moderate
Erber et al, 2021	Yes	Yes	Yes	Yes	Yes	Yes	Yes	Yes	Unclear	Yes	Low
Ferrari et al, 2021	Yes	Yes	Yes	Yes	Unclear	Yes	Yes	Yes	Unclear	N/A	Low
Focosi et al, 2022	Yes	Yes	Yes	Unclear	Unclear	No	Unclear	Yes	No	N/A	High
Franchini et al, 2022	Yes	Yes	Yes	Unclear	Unclear	Yes	Yes	Yes	Yes	Yes	Low
Furlan et al, 2021	Yes	Yes	Yes	Yes	Unclear	Yes	Yes	Yes	Unclear	N/A	Low
Gachoud 1 et al, 2022	Yes	Yes	Yes	Yes	Unclear	Yes	Yes	Yes	No	Yes	Low
Gachoud 2 et al, 2022	Yes	Yes	Yes	Yes	Unclear	No	No	Unclear	No	No	High
Gentile et al, 2024	Yes	Yes	Yes	Yes	Yes	Yes	Yes	Yes	Yes	Yes	Low
Gharbharan et al, 2021	Yes	Yes	Yes	Yes	Unclear	Yes	Yes	Yes	Unclear	N/A	Low
Hueso et al, 2020	Yes	Yes	Yes	Yes	Yes	Yes	Yes	Yes	Unclear	N/A	Low
Huygens et al, 2023	Yes	Yes	Yes	Yes	Yes	Yes	Yes	Yes	No	N/A	Low
Kenig et al, 2021	Yes	Yes	Yes	Yes	Unclear	Yes	Yes	Yes	Yes	N/A	Low
Kluger et al, 2021	Yes	Yes	Yes	Unclear	Unclear	Yes	Yes	Yes	No	N/A	Moderate
Kremer et al, 2021	Yes	Yes	Yes	Unclear	Unclear	Yes	Yes	Yes	No	Yes	Low
Ljungquist et al, 2021	Yes	Yes	Yes	Yes	Yes	Yes	Yes	Yes	Yes	Yes	Low
Martínez-Barranco et al, 2021	Yes	Yes	Yes	Yes	Unclear	Yes	Yes	Yes	Yes	N/A	Low
Nissen-Meyer et al, 2023	Yes	Unclear	Unclear	Yes	Yes	Yes	Yes	Yes	Yes	Yes	Low
Oliva et al, 2022	Yes	Yes	Yes	Yes	Yes	Yes	Yes	Yes	Yes	N/A	Low
Prasad et al, 2021	Yes	Yes	Yes	Unclear	Unclear	Yes	Yes	Yes	Unclear	N/A	Moderate
Ripoll et al, 2022	Yes	Yes	Yes	Yes	Unclear	Yes	Yes	Yes	Yes	N/A	Low
Rüfenacht et al, 2022	Yes	Yes	Yes	Yes	Yes	Yes	Yes	Yes	Unclear	N/A	Low

Q1: Were there clear criteria for inclusion in the case series? Q2: Was the condition measured in a standard, reliable way for all participants included in the case series? Q3: Were valid methods used for identification of the condition for all participants included in the case series? Q4: Did the case series have consecutive inclusion of participants? Q5: Did the case series have complete inclusion of participants? Q6: Was there clear reporting of the demographics of the participants in the study? Q7: Was there clear reporting of clinical information of the participants? Q8: Were the outcomes or follow up results of cases clearly reported? Q9: Was there clear reporting of the presenting site(s)/clinic(s) demographic information? Q10: Was statistical analysis appropriate?
Overall risk of bias was scored in the following way: ≤49% yes = high risk of bias, 50-69% yes = moderate risk of bias, ≥70% yes = low risk of bias.

Table S8. Risk of bias assessment of case reports.

Study	Risk of bias assessment tool questions								Overall risk
	Q1	Q2	Q3	Q4	Q5	Q6	Q7	Q8	
Adedoyin et al, 2021	Yes	Yes	Unclear	Unclear	Unclear	Yes	N/A	Yes	Moderate
Aviv et al, 2021	Yes	No	Yes	Yes	Unclear	Yes	N/A	Unclear	Moderate
Baang et al, 2020	Yes	Yes	Yes	Yes	No	Yes	N/A	Yes	Low
Baek et al, 2023	Yes	Yes	Yes	Yes	Yes	Yes	Yes	Yes	Low
Balashov et al, 2021	Yes	Yes	Yes	Yes	Yes	Yes	N/A	Yes	Low
Basheer et al, 2021	No	No	Yes	Yes	Yes	Yes	N/A	Unclear	Moderate
Belcari et al, 2022	Yes	Yes	Yes	Yes	Yes	Yes	N/A	Yes	Low
Bruiners et al, 2022	Yes	Yes	Yes	Yes	Yes	Yes	N/A	Unclear	Low
Casarola et al, 2021	Yes	Yes	Yes	Yes	Unclear	Yes	N/A	Yes	Low
Chen et al, 2024	Yes	Yes	Yes	Yes	Yes	Yes	N/A	Yes	Low
Clark et al, 2020	Yes	Yes	Yes	Yes	Yes	Yes	Yes	Yes	Low
Colombo et al, 2022	Yes	Yes	Yes	Yes	Yes	Yes	N/A	Yes	Low
Garcia-Vidal et al, 2022	Unclear	Yes	Yes	Yes	Unclear	No	N/A	Yes	Moderate
Gibson et al, 2021	Yes	Yes	Yes	Yes	Yes	Yes	N/A	Yes	Low
Helleberg et al, 2020	Unclear	Yes	Yes	Yes	Yes	Yes	N/A	Yes	Low
Honjo et al, 2020	Yes	Yes	Yes	Yes	Yes	Yes	N/A	Yes	Low
Hughes et al, 2021	Yes	Unclear	Yes	Yes	Yes	Yes	N/A	Yes	Low
Jassem et al. 2021	Yes	Yes	Yes	Yes	Yes	Yes	N/A	Yes	Low
Karaolidou et al, 2021	Yes	Yes	Yes	Yes	Yes	Yes	N/A	Yes	Low
Khatamzas et al, 2022	Unclear	Yes	No	Unclear	Unclear	No	N/A	Unclear	High
Lancman et al, 2020	Yes	Yes	Yes	Yes	Yes	Yes	N/A	Unclear	Low
Librizzi et al, 2024	Yes	Yes	Yes	Yes	Unclear	Yes	N/A	Yes	Low
Malsy et al, 2020	Yes	Yes	Yes	Yes	Yes	Yes	N/A	Unclear	Low
Martínez-Chinchilla et al, 2022	Yes	Yes	Yes	Yes	Unclear	Yes	N/A	Yes	Low
Martinot et al, 2020	Yes	Yes	Yes	Yes	Yes	Yes	N/A	No	Low
McKemey et al, 2021	Yes	Yes	Yes	Yes	Yes	Yes	N/A	Yes	Low
Moore et al, 2020	Yes	Unclear	Yes	Yes	Yes	Yes	N/A	Yes	Low
Moutinho-Pereira et al, 2021	Yes	Yes	Yes	Yes	Yes	Yes	N/A	Yes	Low
Niu et al, 2020	Yes	Yes	Yes	Yes	Yes	Yes	N/A	Yes	Low

Ordaya et al, 2022	Yes	Yes	Yes	Yes	Unclear	Yes	N/A	Yes	Low
Ormazabal Vélez et al, 2021	Yes	Yes	Yes	Yes	Yes	Yes	Yes	Yes	Low
Reuken et al, 2021	Yes	Yes	Yes	Yes	Unclear	Yes	N/A	Yes	Low
Rnjak et al, 2021	Yes	Yes	Yes	Yes	Yes	Yes	N/A	Yes	Low
Rodriguez-Pla et al, 2021	Yes	Yes	Yes	Yes	Unclear	Yes	N/A	Yes	Low
Schenker et al, 2021	Yes	Yes	Yes	Yes	Yes	Yes	N/A	Yes	Low
Sepulcri et al, 2021	Yes	Yes	Yes	Yes	Unclear	Yes	N/A	Yes	Low
Seth-Smith et al, 2023	Unclear	Yes	Yes	Yes	Yes	Yes	N/A	Yes	Low
Spinicci et al, 2022	Yes	Yes	Yes	Yes	Yes	Yes	N/A	Yes	Low
Szwebel et al, 2021	No	Yes	Yes	Yes	Yes	Yes	N/A	Yes	Low
Taha et al, 2021	Yes	Yes	Yes	Yes	Unclear	Yes	N/A	Yes	Low
Tomisti et al, 2023	Yes	Yes	Yes	Yes	Yes	Yes	Yes	Yes	Low
Villaseñor-Echavarri et al, 2023	Yes	Yes	Yes	Yes	Yes	Yes	N/A	Yes	Low
Wright et al, 2021	Yes	Yes	Yes	Yes	Yes	Yes	N/A	Yes	Low
Zhu et al, 2023	Yes	Yes	Yes	Yes	Unclear	Yes	N/A	Yes	Low
Zimmerli et al, 2021	Yes	Yes	Yes	Yes	Unclear	Yes	N/A	Yes	Low

Q1: Were patient’s demographic characteristics clearly described? Q2: Was the patient’s history clearly described and presented as a timeline? Q3: Was the current clinical condition of the patient on presentation clearly described? Q4: Were diagnostic tests or assessment methods and the results clearly described? Q5: Was the intervention(s) or treatment procedure(s) clearly described? Q6: Was the post-intervention clinical condition clearly described? Q7: Were adverse events (harms) or unanticipated events identified and described? Q8: Does the case report provide takeaway lessons?
Overall risk of bias was scored in the following way: ≤49% yes = high risk of bias, 50-69% yes = moderate risk of bias, ≥70% yes = low risk of bias.

Table S9. World Health Organization (WHO) disease severity scale for COVID-19.

WHO ordinal clinical severity scale	
0	No clinical or virological evidence of infection
1	Ambulatory, no activity limitation
2	Ambulatory, activity limitation
3	Hospitalized, no oxygen therapy
4	Hospitalized, oxygen mask or nasal prongs
5	Hospitalized, noninvasive mechanical ventilation or high-flow nasal cannula
6	Hospitalized, intubation and invasive mechanical ventilation (IMV)
7	Hospitalized, IMV + additional support such as pressors or extracardiac membranous oxygenation
8	Death

Table S10. PRISMA Individual Participant Data meta-analysis checklist 2020.

PRISMA-IPD Section/topic	Item No	Checklist item	Reported on page
**Title**			
Title	1	Identify the report as a systematic review and meta-analysis of individual participant data.	Title
**Abstract**			
Structured summary	2	Provide a structured summary including as applicable:	Abstract
		**Background**: state research question and main objectives, with information on participants, interventions, comparators and outcomes.	
		**Methods**: report eligibility criteria; data sources including dates of last bibliographic search or elicitation, noting that IPD were sought; methods of assessing risk of bias.	
		**Results**: provide number and type of studies and participants identified and number (%) obtained; summary effect estimates for main outcomes (benefits and harms) with confidence intervals and measures of statistical heterogeneity. Describe the direction and size of summary effects in terms meaningful to those who would put findings into practice.	
		**Discussion:** state main strengths and limitations of the evidence, general interpretation of the results and any important implications.	
		**Other:** report primary funding source, registration number and registry name for the systematic review and IPD meta-analysis.	
**Introduction**			
Rationale	3	Describe the rationale for the review in the context of what is already known.	Introduction
Objectives	4	Provide an explicit statement of the questions being addressed with reference, as applicable, to participants, interventions, comparisons, outcomes and study design (PICOS). Include any hypotheses that relate to particular types of participant-level subgroups.	Introduction
**Methods**			
Protocol and registration	5	Indicate if a protocol exists and where it can be accessed. If available, provide registration information including registration number and registry name. Provide publication details, if applicable.	Protocol and registration
Eligibility criteria	6	Specify inclusion and exclusion criteria including those relating to participants, interventions, comparisons, outcomes, study design and characteristics (e.g. years when conducted, required minimum follow-up). Note whether these were applied at the study or individual level i.e. whether eligible participants were included (and	Eligibility criteria

		ineligible participants excluded) from a study that included a wider population than specified by the review inclusion criteria. The rationale for criteria should be stated.	
Identifying studies - information sources	7	Describe all methods of identifying published and unpublished studies including, as applicable: which bibliographic databases were searched with dates of coverage; details of any hand searching including of conference proceedings; use of study registers and agency or company databases; contact with the original research team and experts in the field; open adverts and surveys. Give the date of last search or elicitation.	Information sources and search
Identifying studies - search	8	Present the full electronic search strategy for at least one database, including any limits used, such that it could be repeated.	Information sources and search
Study selection processes	9	State the process for determining which studies were eligible for inclusion.	Study selection and data collection processes
Data collection processes	10	Describe how IPD were requested, collected and managed, including any processes for querying and confirming data with investigators. If IPD were not sought from any eligible study, the reason for this should be stated (for each such study).	Study selection and data collection processes
		If applicable, describe how any studies for which IPD were not available were dealt with. This should include whether, how and what aggregate data were sought or extracted from study reports and publications (such as extracting data independently in duplicate) and any processes for obtaining and confirming these data with investigators.	
Data items	11	Describe how the information and variables to be collected were chosen. List and define all study level and participant level data that were sought, including baseline and follow-up information. If applicable, describe methods of standardising or translating variables within the IPD datasets to ensure common scales or measurements across studies.	Study selection and data collection processes
IPD integrity	A1	Describe what aspects of IPD were subject to data checking (such as sequence generation, data consistency and completeness, baseline imbalance) and how this was done.	Study selection and data collection processes
Risk of bias assessment in individual studies.	12	Describe methods used to assess risk of bias in the individual studies and whether this was applied separately for each outcome. If applicable, describe how findings of IPD checking were used to inform the assessment. Report if and how risk of bias assessment was used in any data synthesis.	Ethical Approval and Quality Assessment

Specification of outcomes and effect measures	13	State all treatment comparisons of interests. State all outcomes addressed and define them in detail. State whether they were pre-specified for the review and, if applicable, whether they were primary/main or secondary/additional outcomes. Give the principal measures of effect (such as risk ratio, hazard ratio, difference in means) used for each outcome.	Study selection and data collection processes
Synthesis methods	14	Describe the meta-analysis methods used to synthesise IPD. Specify any statistical methods and models used. Issues should include (but are not restricted to):<br>• Use of a one-stage or two-stage approach.<br>• How effect estimates were generated separately within each study and combined across studies (where applicable).<br>• Specification of one-stage models (where applicable) including how clustering of patients within studies was accounted for.<br>• Use of fixed or random effects models and any other model assumptions, such as proportional hazards.<br>• How (summary) survival curves were generated (where applicable).<br>• Methods for quantifying statistical heterogeneity (such as I^2 and t^2).<br>• How studies providing IPD and not providing IPD were analysed together (where applicable).<br>• How missing data within the IPD were dealt with (where applicable).	Study selection and data collection processes<br><br>&<br><br>Statistical analysis
Exploration of variation in effects	A2	If applicable, describe any methods used to explore variation in effects by study or participant level characteristics (such as estimation of interactions between effect and covariates). State all participant-level characteristics that were analysed as potential effect modifiers, and whether these were pre-specified.	N/A
Risk of bias across studies	15	Specify any assessment of risk of bias relating to the accumulated body of evidence, including any pertaining to not obtaining IPD for particular studies, outcomes or other variables.	Ethical Approval and Quality Assessment
Additional analyses	16	Describe methods of any additional analyses, including sensitivity analyses. State which of these were pre-specified.	Statistical analysis
**Results**			
Study selection and IPD obtained	17	Give numbers of studies screened, assessed for eligibility, and included in the systematic review with reasons for exclusions at each stage. Indicate the number of studies and participants for which IPD were sought and for which IPD were obtained. For those studies where IPD were not available, give the numbers of studies and participants for which aggregate data were available. Report reasons for non-availability of IPD. Include a flow diagram.	Study selection and characteristics

Study characteristics	18	For each study, present information on key study and participant characteristics (such as description of interventions, numbers of participants, demographic data, unavailability of outcomes, funding source, and if applicable duration of follow-up). Provide (main) citations for each study. Where applicable, also report similar study characteristics for any studies not providing IPD.	Study selection and characteristics
IPD integrity	A3	Report any important issues identified in checking IPD or state that there were none.	Study selection and characteristics
Risk of bias within studies	19	Present data on risk of bias assessments. If applicable, describe whether data checking led to the up-weighting or down-weighting of these assessments. Consider how any potential bias impacts on the robustness of meta-analysis conclusions.	Risk assessment & Tables S4-S8
Results of individual studies	20	For each comparison and for each main outcome (benefit or harm), for each individual study report the number of eligible participants for which data were obtained and show simple summary data for each intervention group (including, where applicable, the number of events), effect estimates and confidence intervals. These may be tabulated or included on a forest plot.	Exploratory analysis of individual participant data
Results of syntheses	21	Present summary effects for each meta-analysis undertaken, including confidence intervals and measures of statistical heterogeneity. State whether the analysis was pre-specified, and report the numbers of studies and participants and, where applicable, the number of events on which it is based.	N/A
		When exploring variation in effects due to patient or study characteristics, present summary interaction estimates for each characteristic examined, including confidence intervals and measures of statistical heterogeneity. State whether the analysis was pre-specified. State whether any interaction is consistent across trials.	
		Provide a description of the direction and size of effect in terms meaningful to those who would put findings into practice.	
Risk of bias across studies	22	Present results of any assessment of risk of bias relating to the accumulated body of evidence, including any pertaining to the availability and representativeness of available studies, outcomes or other variables.	Risk assessment & Tables S4-S8
Additional analyses	23	Give results of any additional analyses (e.g. sensitivity analyses). If applicable, this should also include any analyses that incorporate aggregate data for studies that do not have IPD. If applicable, summarise the main meta-analysis results following the inclusion or exclusion of studies for which IPD were not available.	Exploratory analysis of individual participant data

**Discussion**			
Summary of evidence	24	Summarize the main findings, including the strength of evidence for each main outcome.	Discussion
Strengths and limitations	25	Discuss any important strengths and limitations of the evidence including the benefits of access to IPD and any limitations arising from IPD that were not available.	Discussion
Conclusions	26	Provide a general interpretation of the findings in the context of other evidence.	Discussion
Implications	A4	Consider relevance to key groups (such as policy makers, service providers and service users). Consider implications for future research.	Discussion
**Funding**			
Funding	27	Describe sources of funding and other support (such as supply of IPD), and the role in the systematic review of those providing such support.	Funding

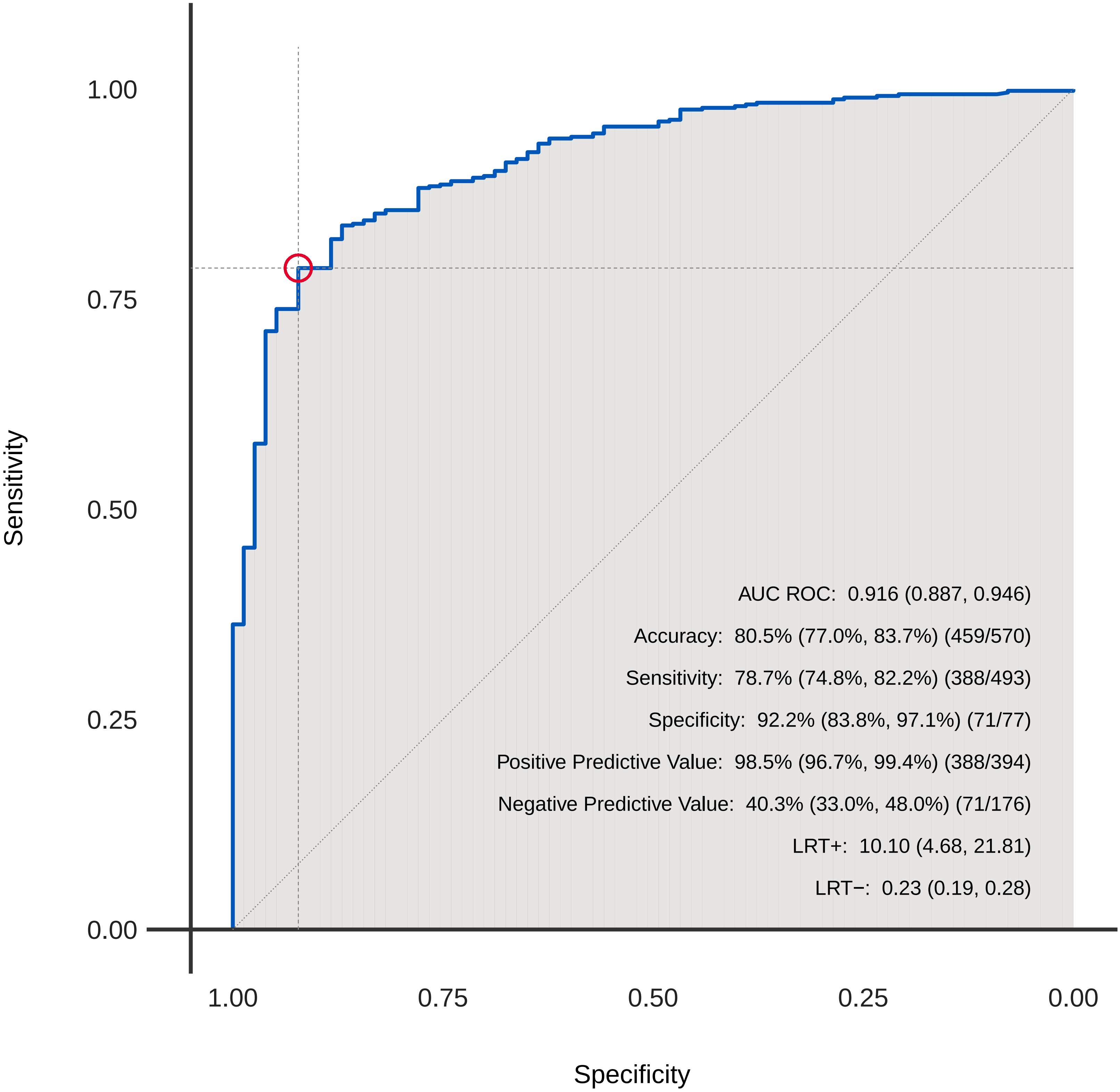

Figure S1. Receiver operating characteristic curve from final XGBoost model for predicting 60-day survival. Performance is summarized at a threshold of 0.83 or higher, which was chosen based on the Youden index. AUC, area under the receiver operating characteristic curve; LRT, likelihood ratio test.
